# Does fluoride influence oviposition of *Anopheles stephensi* in stored water habitats in an urban setting?

**DOI:** 10.1186/s12936-016-1594-x

**Published:** 2016-11-09

**Authors:** Shalu Thomas, Sangamithra Ravishankaran, N. A. Johnson Amala Justin, Aswin Asokan, T. Maria Jusler Kalsingh, Manu Thomas Mathai, Neena Valecha, Alex Eapen

**Affiliations:** 1 IDVC Field Unit, National Institute of Malaria Research (ICMR), NIE Campus, 2nd Main Road, TNHB, Ayapakkam, Chennai, 600 077 India; 2 Department of Zoology, Madras Christian College, Tambaram, Chennai, 600 059 India; 3 National Institute of Malaria Research (ICMR), Sector 8, Dwarka, New Delhi, 110 077 India

**Keywords:** Physico-chemical factors, Breeding habitats, *Anopheles stephensi*, Fluoride

## Abstract

**Background:**

The physico-chemical characteristics of lentic aquatic habitats greatly influence mosquito species in selecting suitable oviposition sites; immature development, pupation and adult emergence, therefore are considerations for their preferred ecological niche. Correlating water quality parameters with mosquito breeding, as well as immature vector density, are useful for vector control operations in identifying and targeting potential breeding habitats.

**Methods:**

A total of 40 known habitats of *Anopheles stephensi*, randomly selected based on a vector survey in parallel, were inspected for the physical and chemical nature of the aquatic environment. Water samples were collected four times during 2013, representing four seasons (i.e., ten habitats per season). The physico-chemical variables and mosquito breeding were statistically analysed to find their correlation with immature density of *An. stephensi* and also co-inhabitation with other mosquito species.

**Results:**

*Anopheles stephensi* prefer water with low nitrite content and high phosphate content. Parameters such as total dissolved solids, electrical conductivity, total hardness, chloride, fluoride and sulfate had a positive correlation in habitats with any mosquito species breeding (p < 0.05) and also in habitats with *An. stephensi* alone breeding. Fluoride was observed to have a strong positive correlation with immature density of *An. stephensi* in both overhead tanks and wells.

**Conclusion:**

Knowledge of larval ecology of vector mosquitoes is a key factor in risk assessment and for implementing appropriate and sustainable vector control operations. The presence of fluoride in potential breeding habitats and a strong positive correlation with *An. stephensi* immature density is useful information, as fluoride can be considered an indicator/predictor of vector breeding. Effective larval source management can be focussed on specified habitats in vulnerable areas to reduce vector abundance and malaria transmission.

**Electronic supplementary material:**

The online version of this article (doi:10.1186/s12936-016-1594-x) contains supplementary material, which is available to authorized users.

## Background

Mosquitoes exploit almost all types of lentic aquatic environments for oviposition [1]. Immature *Anopheles* thrive in a variety of aquatic ecosystems, such as fresh, brackish waters found in rural, coastal and urban areas. Water quality of breeding habitat is an important determinant of whether or not the female mosquitoes will lay their eggs and the resulting immature stages will successfully complete their development to adults [2]. Characteristics of aquatic habitats are reported to influence the preferences of oviposition, possibility of hatching, immature development, pupation, and adult emergence, thus specifying the niche of a mosquito species [3]. Consequently breeding habitat characteristics could influence adult productivity and if the mosquito is of epidemiological importance, it will have a strong impact on the disease transmission [4]. Selection of an appropriate aquatic medium for oviposition could be an innate behavioural characteristic of a mosquito species. What drives a mosquito to select its ovipositional site from an array of aquatic habitats is yet to be fully ascertained and this information would be of paramount importance for focussing intervention operations to target potential vector breeding habitats, thereby reducing manpower and expenditure.

Information on the physico-chemical properties of the breeding habitat, a potential key element for larval surveillance could help in the implementation of better vector control programmes. Effective larval source management (LSM) requires a thorough knowledge of the breeding ecology of the mosquito species, its ovipositional preference, spatial and temporal distribution of the breeding sites besides, physical, chemical and biological characteristics of the habitats. Physico-chemical characteristics of the mosquito breeding habitat such as pH, optimum temperature, total suspended solids, total dissolved solids, electrical conductivity, have an impact on larval development and survival [5]. Furthermore, temperature, salinity, carbonates and nitrates have been shown to correlate with the presence or development of quality of *Anopheles* larvae in pools [6]. Physico-chemical parameters of *Anopheles* breeding habitats in Iran indicated that there was a significant relationship between water temperature, conductivity, total alkalinity, sulfate, chloride, and *Anopheles* species distribution and abundance [7].

*Anopheles stephensi*, the vector responsible for urban malaria in Chennai, India, breeds mainly in clean/clear water, such as overhead tanks, wells, cisterns, roof gutters, curing pits in construction sites, fountains and ornamental tanks. Besides other man-made habitats, such as barrels or drums, sumps or underground tanks, and plastic pots/containers also contributes to enhanced mosquito/vector breeding [8]. Quantifying water quality in *Anopheles* breeding habitats may give more insight into its breeding profile, particularly in urban settings. It was observed that a larger proportion of OHTs support breeding of *An. stephensi*, compared to wells and other breeding habitats. Nevertheless, it was unclear whether this difference was because of differences in the abiotic factor, such as water quality, or biotic, such as co-inhabitation of other mosquito species with the urban vector [8]. Since relatively little information is available on habitat selection and physico-chemical factors determining oviposition behaviour of *An. stephensi* in field settings, the present study aimed to find the relationship between the physico-chemical factors of breeding habitats, such as OHTs and wells, with the occurrence and abundance of *An. stephensi*, in a malarious area of Besant Nagar in Chennai of Peninsular India, for effective vector monitoring and disease control strategy.

## Methods

### Study site and sample collection

**Fig. 1:**
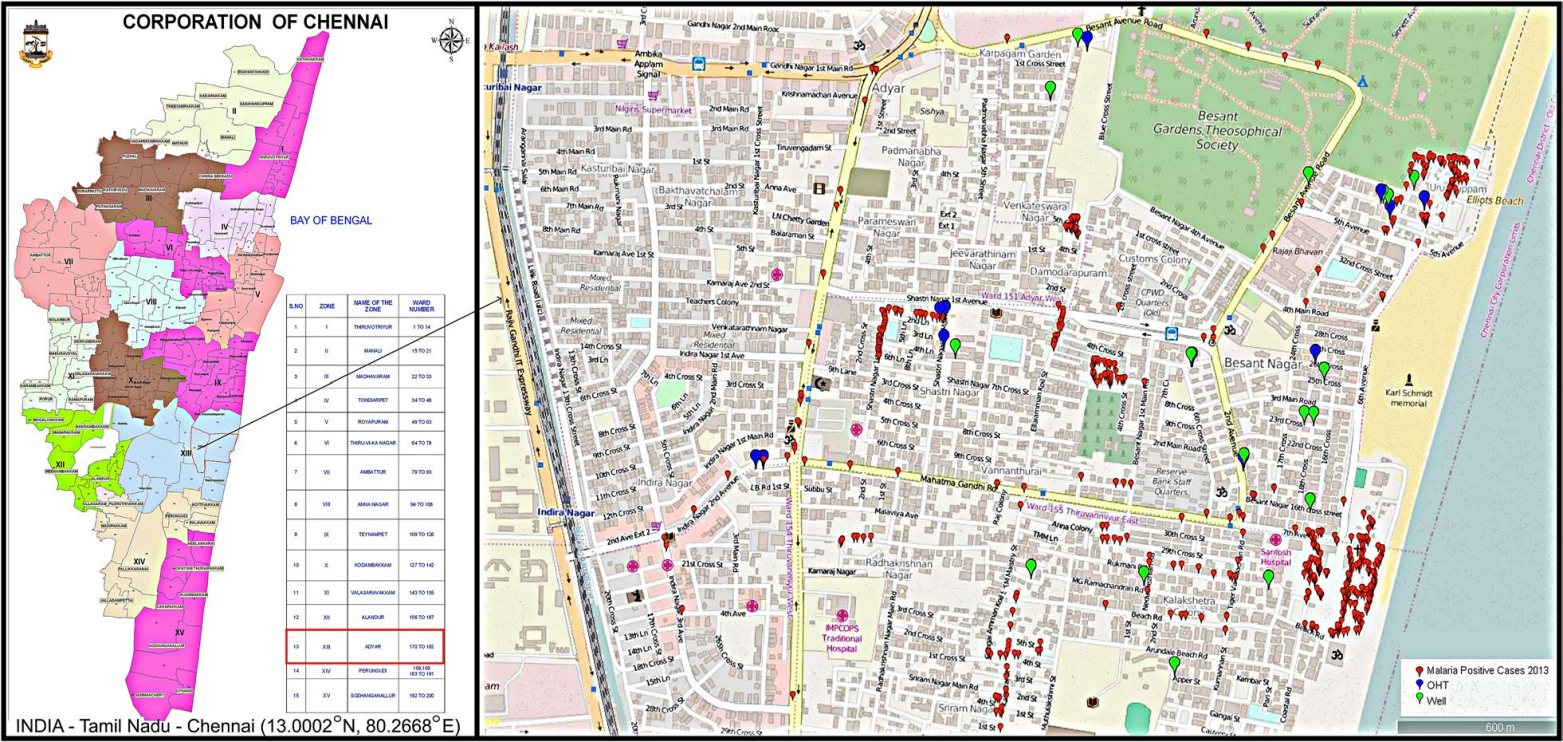
Study area indicating malaria prevalence during 2013 and water sampling sites

Besant Nagar is a residential area with slums, on the seashore in southeastern Chennai (13.0002°N, 80.2668°E), characterized by its meso-endemic, perennial transmission of malaria, predominantly *Plasmodium vivax*, by the Asiatic urban malaria vector, *An. stephensi*. In the present study, potential *An. stephensi* habitats, OHTs and wells were randomly selected from each season of our yearlong weekly study of 20 sentinel and 20 random sites conducted in parallel in the same area [8], to evaluate physico-chemical properties of the habitat and its relation to occurrence and abundance of mosquito breeding (Fig. 1).

A total of 40 habitats i.e., ten habitats (five OHTs and five wells) were randomly selected from each season of a longitudinal study carried out in parallel to check the breeding pattern of vector mosquitoes [8]. Water samples were collected four times during April 2013 to March 2014, representing four seasons: April (summer), July (pre-monsoon), December (winter), and February (post-monsoon).

Among the possible/measurable physico-chemical parameters, physical parameters, such as turbidity, electrical conductivity (EC), total dissolved solids (TDS), and chemical parameters, such as pH, alkalinity, total hardness, nitrites, nitrates, chlorides, fluorides, sulfate biochemical oxygen demand (BOD) dissolved oxygen (DO), were selected for analysis, since they have been reported to have correlation with mosquito breeding [7, 9]. The habitats were also observed for the presence or absence of direct sunlight or shade, colour, and temperature of water at the time of inspection. Habitat particulars such as whether they were perennial (water stored throughout the year), temporary (occasionally filled/retained depending on need), open (with gaps allowing entry of mosquitoes or completely mosquito-proofed leaving no gaps), were considered from a longitudinal study carried out in parallel [8]. Immature collection in wells were done using ‘well nets’ (conical drop nets 20 cm in diameter and 10 cm deep, that were lowered into the well on a string), while ladles with a volume of 250 mL were used for OHTs, performing four dips per sampling occasion [10–12]. Collected immatures were identified, scored instar-wise and immature density was calculated as the number of immature/dip [8]. *Anopheles* immatures were emerged and identified to the species level using standard morphological identification keys [13, 14]. The physical parameters such as habitat positivity for *Anopheles* immatures (presence or absence of *Anopheles* immatures in the habitat), immature count (instar-wise count of *Anopheles* larvae and pupal count), appearance of water (whether the water was clear or turbid), colour (whether the water seemed colourless or yellowish), sun/shade (whether the water in the habitat is exposed to direct sun or it is shaded by the presence of vegetation or any other covering), open/closed (whether the habitat is closed with a proper lid/covering which does not allow the entry of mosquito) were recorded on site by visual observation whereas temperature was measured using thermometer at the time of inspection. Turbidity, pH, TDS, EC and other chemical parameters were measured in a laboratory of Tamil Nadu Water Supply and Drainage Board (TWAD), Water Analysis Department, an ISO 9001: 2008 certified laboratory for ‘Testing of physical, chemical & bacteriological parameters for water & waste water, testing of treatment chemicals and training in water analysis’.

Water was taken from middle of the source or habitat at mid-depth using a steel bucket of 3 L capacity for wells and a 250-mL capacity dipper in the case of OHTs. The water was then carefully transferred to 2 L sample bottle consisting of an outer screw cap and an inner lid after rinsing with the water to be sampled. The sample bottle was filled to the brim without any air bubbles, labelled with date, time of collection, habitat type and location. They were then transported to the laboratory directly from the field site for immediate analysis. Water sampling was done as per standard sampling procedures and the physico-chemical parameters were analysed in the TWAD laboratory following the methods described by American Public Health Association [15]. The abiotic parameters were analysed based on the presence and absence of *Anopheles* immatures in the habitat at the time of inspection. The minimum, maximum and mean values of the parameters along with the standard deviation and range were calculated.

Student *t* test was applied to find whether the physico-chemical parameters significantly differentiated the habitats with and without mosquito breeding. Mann–Whitney U test was performed to find the significant difference of physico-chemical parameters between habitats where *Anopheles* bred alone and habitats where *Anopheles* were found co-inhabiting with other mosquitoes. Further, Pearson correlation was performed to find the correlation between immature density and physico-chemical parameters in habitats where *Anopheles* bred with other mosquitoes and with habitats where *Anopheles* bred alone, in wells and OHTs. In order to find the influence of all physico-chemical parameters on immature density, simple linear regression model was performed. Statistical analyses were done using SPSS software version 17. The figures, which represent the scatter plot matrix of physico-chemical parameters with significant correlation, were generated using R software version 3.1. Institutional ethical clearance of the project was obtained (ECR/NIMR/EC/2010/100).

## Results

### Mosquito breeding composition and immature density

Immatures of *An. stephensi* alone or in co-inhabitation with other mosquito species were found in 26 of the 40 habitats sampled. The habitats where *An. stephensi* were found alone were: 12 habitats (eight OHTs and four wells); There were ten habitats (nine wells and one OHT) with mixed population of *Anopheles* and *Culex* immatures; another habitat (one well) co-inhabited with *An. stephensi* and *Aedes* immatures; three habitats (two wells and one cistern) with mixed population of *An. stephensi*, *Culex* and *Aedes* immatures. Fourteen breeding habitats had no mosquito breeding at the time of inspection. The immature density of *Anopheles* species ranged from one to 31 per dip with *An. stephensi* breeding alone. *Anopheles* + *Culex* co-inhabited habitats had one to 25 per dip, whereas in *Anopheles* + *Aedes* + *Culex* breeding habitats, the corresponding immature densities were three to ten per dip. The per-dip immature density in habitats with *Anopheles* + *Aedes* breeding was four. Though the objective of the study was to find any correlations between physico-chemical variables and *An. stephensi*, other mosquito species were also encountered while sampling. *Anopheles subpictus* was observed in OHTs and wells, whereas *Culex quinquefasciatus*, *Culex gelidus* and *Aedes aegypti* were the other species found in wells. All the habitats with open structures were located in shaded areas, except one which was directly exposed to sunlight. The water temperature ranged from 27 to 31 °C at the time of inspection.

### Comparison of physico-chemical parameters in habitats with and without mosquito breeding

The physical and chemical parameters from the inspected habitats were analysed statistically (Table 1). The range of values for temperature, turbidity, pH, and fluoride did not vary much among the habitats with and without mosquito breeding. However, the range of TDS, EC, total hardness, alkalinity, chloride, sulfate, BOD, and DO were found more in habitats with mosquito breeding compared to habitats without breeding, whereas a wider range of nitrate, nitrite and phosphate were found in those habitats without mosquito breeding.

**Table 1:** Physico-chemical parameters of habitats with and without mosquito breeding

Parameters	Habitats with mosquito breeding (n = 26)		Habitats without mosquito breeding (n = 14)	
	Range	Mean ± SD	Range	Mean ± SD
Temperature (°C)	27–30	29.12 ± 1.24	27–31	29 ± 1.36
Turbidity (NT)	0.1–2.3	0.61 ± 0.61	0.1–2.3	0.64 ± 0.57
TDS (mg/L)	139–10,913	1558.76 ± 2820.86	134–5480	1304.14 ± 1554.23
EC (micromho/cm)	198–15,590	2386.03 ± 4036.51	192–7830	1832.92 ± 2238.86
pH	6.82–8.72	7.56 ± 0.5	6.6–8.25	6.6 ± 0.51
Total alkalinity (mg/L)	60–760	202.15 ± 144.53	45–280	179.21 ± 72.13
Total hardness (mg/L)	52–2800	399.34 ± 607.18	64–1020	352.71 ± 338.3
Nitrite (mg/L)	0–1.21	0.11 ± 0.24	0–3.81	0.69 ± 1.33
Nitrate (mg/L)	1–46	12.57 ± 13.62	1–52	19.14 ± 19.3
Chloride (mg/L)	13–4800	552.5 ± 1247.98	11–2500	468.07 ± 700.93
Fluoride (mg/L)	0–0.72	0.22 ± 0.18	0.01–0.62	0.22 ± 0.18
Sulfate (mg/L)	1–310	55.46 ± 85.32	0–231	37.71 ± 67.86
Phosphate (mg/L)	0–1.1	0.34 ± 0.29	0–1.51	0.48 ± 0.4
BOD (mg/L)	1.5–30	6.22 ± 6.83	1–25	5.96 ± 6.64
DO (mg/L)	1.5–20	4.13 ± 3.53	1.4–8	3.61 ± 1.94

The mean values of physical parameters, such as turbidity, TDS, total hardness, total alkalinity and EC besides chemical parameters, such as chloride, sulfate, BOD and DO values were higher in habitats with mosquito breeding compared to those without breeding. In habitats without mosquito breeding, the mean pH was found to be slightly acidic (6.6) whereas habitats with mosquito breeding exhibited alkaline pH (7.56), although both values were around the neutral pH (7). The mean values of phosphate, nitrite and nitrate were found to be comparatively higher in habitats without any mosquito breeding compared to those with breeding. When Student t-test was performed, it was observed that the mean value of nitrite was significantly higher in habitats without breeding of *Anopheles* species (Table 1) and the difference was statistically significant (p = 0.035).

Mann–Whitney U test was performed to compare the selected parameters between two groups (Group A: habitats in which *Anopheles* bred alone, Group B: habitats in which *Anopheles* bred with other mosquito species). When these groups were compared, the mean value of phosphate was significantly higher in Group A (0.49 mg/L) than in Group B (0.21 mg/L) indicating higher phosphate content to favour *Anopheles* breeding but not with other mosquito species (Table 2).

**Table 2:** Analysis of physico-chemical parameters of habitats with *Anopheles stephensi* breeding

Parameters	Breeding habitats			
	*Anopheles stephensi* alone		*Anopheles stephensi* with other mosquito species^a^	
	Range	Mean ± SD	Range	Mean ± SD
Temperature (°C)	27–30	29.58 ± 0.9	27–30	28.71 ± 1.38
Turbidity (NT)	0.1–1.4	0.46 ± 0.48	0.1–2.3	0.75 ± 0.69
TDS (mg/L)	174–10,913	2579.17 ± 3909.29	139–3178	684.14 ± 765.58
EC (micromho/cm)	248–15,590	3687.25 ± 5583.08	198–4540	1270.71 ± 1433.76
pH	6.94–8.49	7.52 ± 0.53	6.82–8.72	7.59 ± 0.5
Total alkalinity (mg/L)	60–440	188.67 ± 107.59	72–760	213.71 ± 173.41
Total hardness (mg/L)	80–2800	619 ± 847.58	52–600	211.07 ± 135.66
Nitrite (mg/L)	0–0.37	0.1 ± 0.15	0–1.21	0.13 ± 0.31
Nitrate (mg/L)	1–46	17.17 ± 15.27	1–41	8.64 ± 11.11
Chloride (mg/L)	18–4800	1029 ± 1744.64	13–690	144.07 ± 174.38
Fluoride (mg/L)	0–0.44	0.19 ± 0.14	0–0.72	0.25 ± 0.23
Sulfate (mg/L)	1–239	71.33 ± 91.12	1–310	41.86 ± 80.9
Phosphate (mg/L)	0.07–1.1	0.49 ± 0.31	0–0.82	0.21 ± 0.22
BOD (mg/L)	2–10	4.79 ± 2.62	1.5–30	7.45 ± 8.97
DO (mg/L)	1.5–6.2	3.48 ± 1.52	1.6–20	4.69 ± 4.62

^a^Other mosquito species includes *Anopheles subpictus*, *Culex quinquefasciatus*, *Cx. gelidus* and *Aedes aegypti*

### Correlations of physico-chemical parameters with immature density

**Fig. 2:**
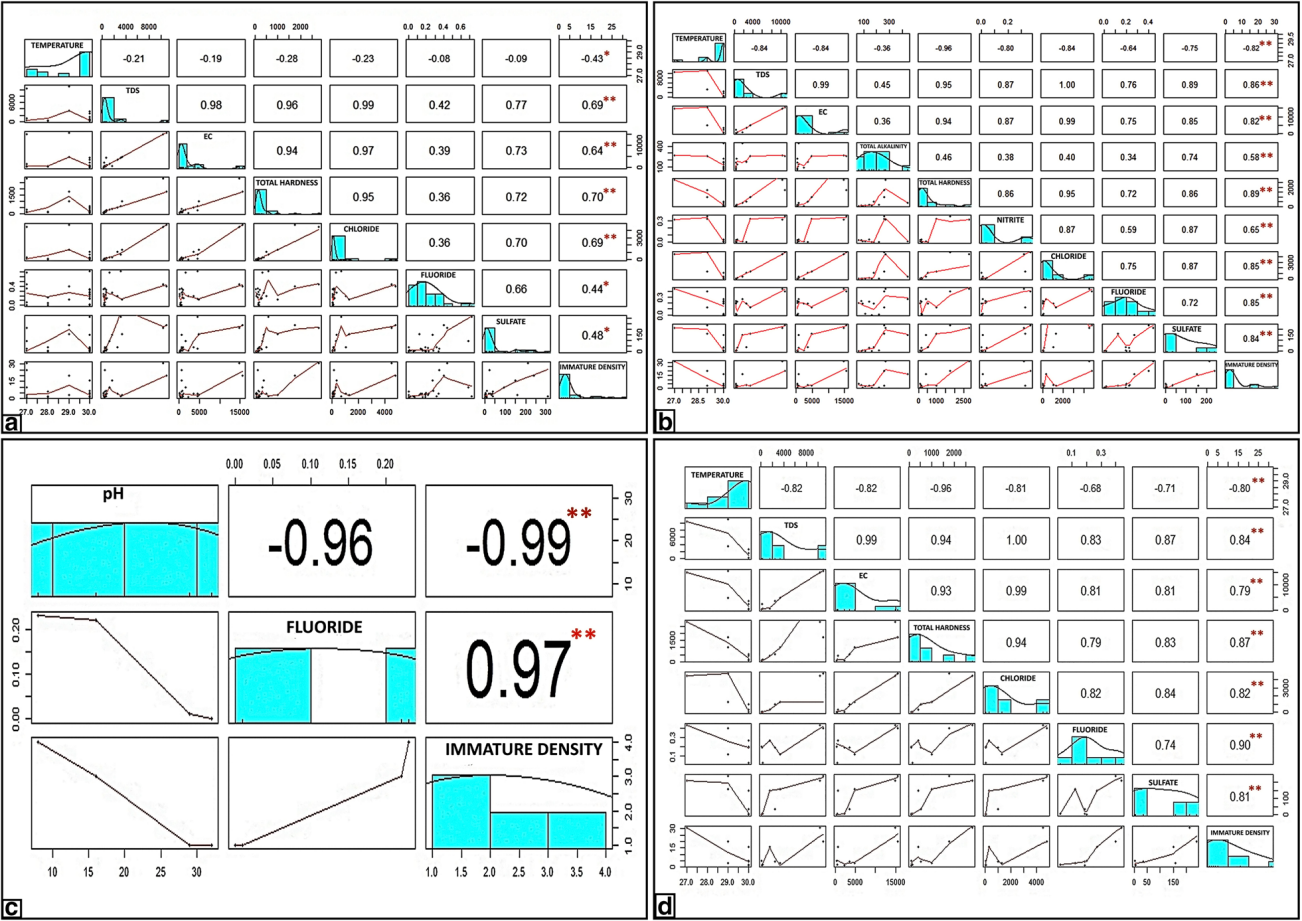
The *scatter plot* matrix of physico-chemical parameters with significant correlation. Correlation of physico-chemical parameters with immature density: **a** in habitats where *Anopheles* co-bred with other mosquito species; **b** in habitats where *Anopheles* bred alone; **c** in wells where *Anopheles* bred alone; **d** in OHTs where *Anopheles* bred alone. The distribution of significant parameters are represented in the *boxes* along the *diagonal line* from extreme *top left* to extreme *bottom right*. Each parameter is plotted against the other below the *diagonal line*. These *boxes* display the bivariate *scatter plots* with a *fitted line*. The corresponding value of the correlation plus the significance level as stars (*p < 0.05; **p < 0.01) are represented in the *boxes* above the diagonal. The* X-axis* values are given for each parameter either at the *top* or *bottom* alternatively in the Figure. Likewise, the* Y-axis* values are also given alternatively either at the *left* or *right*

Pearson correlation was performed to analyse the effect of physico-chemical parameters on immature density of mosquito breeding habitats in general (Fig. 2a). It was found that TDS (r = 0.60), EC (r = 0.60), total hardness (r = 0.61), chloride (r = 0.60), sulfate (r = 0.41), and fluoride (r = 0.30) had significant positive correlation (p < 0.05). Further, when habitats with *Anopheles* breeding alone was considered, alkalinity (r = 0.58) and nitrite (r = 0.65) along with the six parameters that had significant positive correlation with immature density in general, such as TDS (r = 0.86), EC (r = 0.82), total hardness (r = 0.89), chloride (r = 0.85), sulfate (r = 0.84), and fluoride (r = 0.85), comparatively had significantly strong positive correlation (p < 0.05) whereas, temperature (r = 0.82) had negative correlation (Fig. 2b).

When physico-chemical parameters of OHTs and wells were compared using Student *t*-test, it was found that parameters such as temperature, TDS, EC, alkalinity, total hardness, nitrite, nitrate, chloride, fluoride, sulfate, phosphate, and DO were significantly higher in OHTs (p < 0.05). Further, when Pearson correlation was performed to explore the influence of the selected parameters on immature density in wells where *An. stephensi* bred alone, significant positive correlation of immature density with fluoride (r = 0.97) and negative correlation (p < 0.05) with pH (r = 0.99) was observed (Fig. 2c). Likewise, when the data was analysed for OHTs, the parameters TDS (r = 0.84), EC (r = 0.79), total hardness (r = 0.87), chloride (r = 0.82), fluoride (r = 0.90), and sulfate (r = 0.81) had positive correlation (p < 0.05) with immature density, whereas temperature (r = 0.82) was negatively correlated (Fig. 2d).

### Effect of all physico-chemical parameters on immature density

When linear regression model was performed to find the influence of all parameters together on *An. stephensi* immature density, multicollinearity problem was encountered. So, the variables (EC, Alkalinity total, Total hardness, Chloride, Sulfate) with high variance influence factor (VIF > 10) was removed and then the data was re-analysed which showed that fluoride, nitrite and TDS had significant influence on immature density (See Additional file 1: Table S1). Among the three significant parameters, fluoride has the highest regression coefficient value (β = 21.62). The model fit is R^2^ = 0.91 which means 91% of variance was exhibited by this model. Regression equation for immature density was framed based on this model, i.e., Immature density = 21.070 + 0.002(TDS) (15.867*nitrite) + (21.623*fluoride) + Error.

## Discussion

The present study revealed that physico-chemical parameters of water have no impact on mosquito breeding in general except nitrite, which significantly differentiates habitats with mosquito breeding from those without mosquito breeding; nitrite was found to be higher in the latter (Table 1). Although nitrite showed no significant correlation with immature density in habitats with mosquito breeding in general (Fig. 2a), it had a significant positive correlation with immature density in habitats where *Anopheles* bred alone (Fig. 2b). Thus, *An. stephensi* seems to prefer less nitrite content whereas other species such as *Culex* sp. breeds in water with either high or low content of nitrite [16]. Similar observations were made in Kuwait [17] where *An. stephensi* preferred less nitrite content compared to *Culex* sp. and in Ghana where *Anopheles gambiae* preferred much less nitrite content (<0.005 mg/L) [18]. Furthermore, phosphate showed significant difference in habitats with *Anopheles* breeding and when co-inhabitation of *Anopheles* was observed with other mosquito species. It was found to be higher in the former, which explains higher phosphate content favouring *Anopheles* breeding, unlike other mosquito species. A strong positive correlation of phosphate with mosquito larval abundance was observed in Sri Lanka [19]; In Ethiopia it was observed that phosphate was positively correlated with *Anopheles* larvae [20], which is in concordance with the observations of the present study. The high values of phosphate, nitrite and nitrate were reported to be indicators of organic pollution [17, 21].

Six out of 15 parameters analysed in the present study: TDS, EC, total hardness, chloride, fluoride, and sulfate, had significant positive correlation with immature density in habitats with mosquito breeding in general, habitats with *Anopheles* bred alone and in OHTs where *Anopheles* bred alone [7, 22]. In contrast, these six parameters except fluoride did not show any significant correlation in wells where *Anopheles* bred alone whereas, pH had high significant negative correlation.

A number of studies done in various geographical locations around the world (Ethiopia, Kenya, Iran, Delhi) support the fact that temperature is positively correlated with *Anopheles* larval abundance [3, 7, 20, 23]. In Bangladesh [24] temperatures collected from meteorological departments had no correlation with *Anopheles* abundance, which is similar to earlier findings that meteorological data do not give an actual estimate of temperature-dependent traits [25]. In contrast, in the present study, temperature has a strong negative correlation with immature density of *An. stephensi* larvae in OHTs. Since temperature was recorded only at the time of inspection, variations and their impact cannot be taken into consideration as the objectives of the present study were to find any relation or association between physico-chemical variables with occurrence and abundance of *An. stephensi*.

Anopheline immatures have been reported to live in acidic [26] as well as alkaline medium [27]. However, alkaline pH with the range 8–8.5 is considered to be favourable pH for the majority of anophelines [7]. In Iran [7], *An. stephensi* was found with pH ranging 7.1–8.6, which is similar to the present study, where anopheline positive breeding habitats exhibited pH from slightly acidic to alkaline (6.82–8.72). Further, similar to temperature, pH is believed to be positively correlated with *Anopheles* larval density [23, 28], whereas it has been reported that pH had no significant association with *Anopheles* density [29]. The present study revealed that although pH had no correlation with *An. stephensi* density in habitats where it bred alone or with other mosquito species, it had a strong negative correlation with *An. stephensi* immature density in wells where it bred alone.

The highlight of this study is the highly significant positive correlation of fluoride with *Anopheles* immature density. In Ghana *An. gambiae* showed significant positive correlation of fluoride (r = 0.739, p = 0.036) with higher proportions of *An. gambiae* s.s. M-form in larval habitats [18]. In the present study, although fluoride showed significant weak positive correlation with immature density in general, fluoride along with TDS, EC, total hardness, chloride, and sulfate, indicated a very strong positive correlation in habitats where *An. stephensi* bred alone. Moreover, in OHTs fluoride had the highest positive significant correlation value (r = 0.90) among other significant parameters (TDS, EC, total hardness, chloride, sulfate). In the earlier study, OHTs had been discovered as the potential breeding habitat for *An. stephensi* in the same area [8]. Even in wells, fluoride had strong positive correlation with immature density of *An. stephensi* (r = 0.97). Also when all the physico-chemical parameters were analysed together in a habitat with *An. stephensi* breeding, fluoride has significant difference on the immature density. Although it is revealed that higher fluoride content favours *Anopheles* breeding, in the present study it is well within the water quality standards as the range of fluoride concentration (0–0.72 mg/L) observed is not higher than Center for Disease Control (CDC)-recommended optimal fluoride concentration to community water systems, which is 0.7 mg/L [30]. The findings of strong positive correlation between fluoride and immature density of *An. stephensi* irrespective of the nature or type of breeding habitats, such as OHTs and wells, indicates its probable role as an oviposition attractant in luring gravid females of *An. stephensi*, unlike other mosquito species. Hence, fluoride can be considered as a predictor for urban vector breeding and would be an useful indicator for the presence of vector breeding. LSM by using conventional larvicides (Temephos) against urban malaria vectors is traditionally and routinely undertaken in all clear water habitats. In the present study, Urur-Olcott Kuppam, located northeast, and Odaima Nagar, at the southeast, both adjacent to the shore, consists of slum tenements with high prevalence of malaria (Fig. 1). The survey carried out in these two slum areas did not locate potential breeding habitats such as OHTs and wells. However, adjacent areas of these slums had breeding habitats contributing to the abundance and propagation of *An. stephensi* [12].

There are increasingly renewed interests globally to promote/advocate development of appropriate intervention tools to target malaria vector control in aquatic stages, especially in urban settings, where it is operationally difficult to target adult populations due to innumerable, hidden resting habitats where it can evade household repellents. In this context, potential habitats, such as OHTs, which produce significantly more adult vector mosquitoes, may eventually contribute towards malaria transmission and disease burden. It is important to identify such breeding habitats and target them with appropriate LSM to achieve the object of reducing urban malaria. The habitat characteristics observed in the present study can be used to further explore operational ways in which LSM can be focussed against urban malaria vectors. The application of such innovative intervention tools needs to be easy for operational field personnel to effectively identify the most productive potential breeding habitats. This method would ultimately help in arresting vector abundance as a long-term and permanent solution.

The limitation of this study is that the variation in fluoride concentration and vector density was not analysed. The sampling of physico-chemical parameters were comparatively less mainly because the analysis was outsourced and the study objective was limited to the occurrence of *Anopheles* breeding and its possible correlation with the physico-chemical parameters. In future, selection of a few important physio-chemical variables can be analysed to target more habitats to obtain a robust data which helps to develop a mathematical model which may be useful for situation based vector control interventions. Further, a year-long collection of water samples from sentinel and random sites were not carried out to find seasonal variations in physico-chemical parameters and their correlation to mosquito breeding and/or immature *An. stephensi* density. The present study describes the relationship between breeding habitats and physico-chemical parameters, but not the causal association.

## Conclusion

Control of immatures through source reduction and routine application of larvicides are considered to be key intervention tools in eliminating malaria. These measures are intended to reduce transmission of malaria by preventing propagation of vector mosquitoes and subsequently reducing vector-pathogen-human contact. Knowledge of larval vector ecology is a key factor in risk assessment and implementation of effective vector control operations. It may be argued that physico-chemical parameters in anopheline breeding habitats reflects the ovipositional preference or behaviour of these mosquitoes to identify the most appropriate from diverse breeding habitat environments in an urban settlement. Periodic seasonal analysis will help to give a vivid and broader picture of how the various parameters of breeding waters influence mosquito bionomics and population dynamics. The information thus obtained will help in proper planning of intervention measures for vector control programmes.

## Additional file

**Additional file 1: Table S1** Linear regression model for physico-chemical parameters and immature density.

## Abbreviations

OHT: overhead tank
LSM: larval source management
TDS: total dissolved solids
BOD: biological oxygen demand
DO: dissolved oxygen
EC: electrical conductivity
TWAD: Tamil Nadu Water Supply and Drainage Board
CDC: Center for Disease Control
CMWSSB: Chennai Metropolitan Water Supply and Sewerage Board
