# Supplementary material for: Does fluoride influence oviposition of Anopheles stephensi in stored water habitats in an urban setting?
Source: Malar J. 2016 Nov 9;15:549. doi: 10.1186/s12936-016-1594-x (PMC5103410; doi:10.1186/s12936-016-1594-x)
Supplement: Supplementary file 1 — Additional file 1: Table S1 Linear regression model for physico-chemical parameters and immature density. [file 12936_2016_1594_MOESM1_ESM.docx]

**Additional file 1:** Linear regression model for physico-chemical parameters and immature density

| Independent Factors | β | t | p VALUE |
| --- | --- | --- | --- |
| (Constant) | 21.07 | 0.607 | 0.554 |
| Colour | 7.502 | 2.047 | 0.061 |
| Sunlight/Shade | 1.618 | 0.612 | 0.551 |
| Temperature | -1.168 | -1.18 | 0.257 |
| Turbidity | -0.062 | -0.03 | 0.978 |
| TDS | 0.002 | 3.787 | 0.002 |
| pH | 0.569 | 0.181 | 0.859 |
| Nitrite | -15.87 | -2.63 | 0.021 |
| Nitrate | -0.094 | -0.87 | 0.398 |
| Fluoride | 21.623 | 2.605 | 0.022 |
| Phosphate | 3.697 | 0.884 | 0.393 |
| BOD | 0.101 | 0.531 | 0.604 |
| DO | -0.496 | -1.32 | 0.209 |
